# Perioperative evaluation of respiratory impedance using the forced oscillation technique: a prospective observational study

**DOI:** 10.1186/s12871-016-0197-y

**Published:** 2016-07-07

**Authors:** Shoko Nakano, Junko Nakahira, Toshiyuki Sawai, Yosuke Kuzukawa, Junichi Ishio, Toshiaki Minami

**Affiliations:** Department of Anesthesiology, Osaka Medical College, 2-7 Daigaku-machi, Takatsuki, Osaka 569-8686 Japan

**Keywords:** Forced oscillation technique, Respiratory impedance, Ventilator-induced lung injury, Mechanical ventilation, General anesthesia

## Abstract

**Background:**

Intravascular fluid shifts, mechanical ventilation and inhalational anesthetic drugs may contribute to intraoperative lung injury. This prospective observational study measured the changes in respiratory impedance resulting from inhalational anesthesia and mechanical ventilation in adults undergoing transurethral resection of bladder tumors. The components of respiratory impedance (resistance and reactance) were measured using the forced oscillation technique (FOT).

**Methods:**

Respiratory resistance at 5 Hz (R5) and 20 Hz (R20), respiratory reactance at 5 Hz (X5), resonant frequency (Fres) and area of low reactance (ALX) were measured before and immediately after surgery in 30 adults. In addition, preoperative vital capacity (VC), forced vital capacity (FVC) and forced expiratory volume in the first second (FEV1.0) were evaluated using spirometry. All patients were intubated with an endotracheal tube and were mechanically ventilated, with anesthesia maintained with sevoflurane. Pre- and postoperative FOT measurements were compared using Wilcoxon paired rank tests, and the relationships between FOT measurements and preoperative spirometry findings were determined by Spearman’s rank correlation analysis.

**Results:**

Twenty-six patients were included in the final analysis: postoperative FOT could not be performed in four because of postoperative restlessness or nausea. The mean duration of surgery was 47 min. All components of respiratory resistance deteriorated significantly over the course of surgery, with median increases in R5, R20, and R5–R20 of 1.67 cmH_2_O/L/s (*p* < 0.0001), 1.28 cmH_2_O/L/s (*p* < 0.0001) and 0.46 cmH_2_O/L/s (*p* = 0.0004), respectively. The components of respiratory reactance also deteriorated significantly, with X5 decreasing 1.7 cmH_2_O/L/s (*p* < 0.0001), Fres increasing 5.57 Hz (*p* < 0.0001) and ALX increasing 10.51 cmH_2_O/L/s (*p* < 0.0001). There were statistically significant and directly proportional relationships between pre- and postoperative X5 and %VC, %FEV1.0 and %FVC, with inverse relationships between pre- and postoperative Fres and ALX.

**Conclusions:**

All components measured by FOT deteriorated significantly after a relatively short period of general anesthesia and mechanical ventilation. All components of resistance increased. Of the reactance components, X5 decreased and Fres and ALX increased. Pre- and postoperative respiratory reactance correlated with parameters measured by spirometry.

**Trial registration:**

JMA-IIA00136.

## Background

The forced oscillation technique (FOT) is a non-invasive method of measuring respiratory impedance at rest [1, 2]. This technique is based on the external application of a pressure wave to the respiratory system, followed by measurement of the resultant airflow. The FOT has entered routine clinical practice for the evaluation and management of patients with obstructive respiratory diseases, including chronic obstructive pulmonary disease (COPD) and asthma [3–6].

Spirometry remains the most frequent method of evaluating preoperative respiratory function; this method, however, is somewhat effort dependent, as it requires the subject to make a maximal forced expiratory maneuver. Therefore, spirometry tends not to be used for postoperative evaluation. Postoperative respiratory function is generally determined by measuring vital signs, such as peripheral oxygen saturation, and, when appropriate, by arterial blood gas analysis. These methods, however, cannot assess postoperative respiratory deterioration. This difficulty may be overcome by using FOT, which requires minimal patient effort. FOT is based on measuring the relationship between pressure waves applied externally to the respiratory system and the resulting respiratory airflow. Impedance of the respiratory system is defined as the ratio of the amplitude of the pressure wave to the amplitude of the resulting flow wave. The FOT measures the two components of respiratory impedance, respiratory resistance (Rrs) and respiratory reactance (Xrs).

**Fig. 1:**
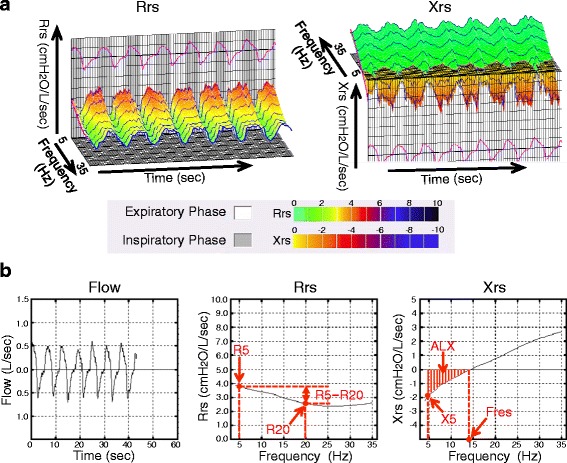
**a** Representative 3D color graph of postoperative respiratory resistance and reactance, measured using the MostGraph-01. The x-, y-, and z-axes show time, respiratory resistance (Rrs) or respiratory reactance (Xrs), and frequency (5–35 Hz), respectively. **b** Representative 2D graph of postoperative respiratory resistance and respiratory reactance measured using the MostGraph-01. Respiratory resistance (Rrs) was measured at 5 Hz (R5) and 20 Hz (R20), and their difference was calculated (R5 − R20; frequency dependence of respiratory resistance). Respiratory reactance (Xrs) was measured at 5 Hz (X5), as were Fres (frequency at a respiratory reactance of 0 cm H_2_O/L/s) and area of low respiratory reactance (ALX, the area made by three lines of frequency 5 Hz, Xrs = 0 cmH_2_O/L/s and the Xrs curve). Rrs and Xrs represent the average values of inspiratory and expiratory phases, respectively

A “broadband” FOT device, the MostGraph-01 (Chest MI, Tokyo, Japan), became commercially available in Japan in 2008 [5, 7] and is widely used in pediatric and allergy clinics [8]. This device can measure respiratory impedance, including Rrs and Xrs, using color 3D imaging [5, 9]. Rrs measured by FOT includes the resistance of the oropharynx, larynx, trachea, large and small airways, lungs and chest wall tissue. By using multiple oscillation frequencies, however, the behaviors of large and peripheral small airways can be distinguished. Sound waves at frequency <15 Hz travel more distally, whereas those >20 Hz are damped out by intermediate sized airways [10]. Rrs measured at 5 Hz (R5) and 20 Hz (R20) are representative of low and high frequency resistance, respectively, with the difference between R5 and R20 (R5 − R20) termed the frequency dependence of Rrs [11]. R5 represents total airway resistance, R20 represents the resistance of the large airways [10], and R5 − R20 can logically reflect the resistance of the small airways. Normal limits for R5 and R20 have not been established. However, a study of the impulse oscillometry system in normal individuals classified resistance oscillation frequencies of ≤2 cmH_2_O/L/s as normal, those between 2 and 3 cmH_2_O/L/sec as borderline, and those ≥3 cmH_2_O/L/s as highly resistant [12, 13]. At lower frequencies, the value of Rrs is greater; consequently, patients with COPD tend to have a greater R5 − R20, which is thought to reflect heterogeneous ventilatory mechanics [14]. Xrs is the imaginary part of respiratory impedance and includes the mass inertial forces of the moving air column, expressed in terms of inertia, and the elastic properties or compliance of the lung periphery, expressed as capacitance. The reactance at 5 Hz (X5) reflects the combined effects of tissue elastance and inertia, although at this lower frequency, the effect of tissue elastance would be predominant. X5 therefore reflects the elastic recoil of the peripheral airways [9, 15]. Because the ability of the lungs to store capacitative energy is primarily manifest in the small airways, X5 can provide important information about the distal/small airways. States that reduce the elasticity of the lungs, such as fibrosis and hyperinflation, make the capacitance more negative, with more negative or higher X5 values [10]. The resonant frequency (Fres) is the frequency at which respiratory reactance equals 0 cmH_2_O/L/s. Fres marks the transition from capacitative dominance at low frequencies to inertial dominance at high frequencies. This also helps conveniently categorize frequencies below Fres as low and above Fres as high, and indicates a frequency at which the total impedance to airflow is completely resistive. Normal Fres is approximately 6–11 Hz. Fres tends to be higher in children, decreases with age and increases in individuals with both obstructive and restrictive diseases [10]. The area of low reactance (ALX) is the area created by three lines (that of frequency 5Hz, Xrs = 0 cmH_2_O/L/s and the Xrs curve). ALX is a useful index associated with respiratory compliance and therefore of the patency of the small airways. ALX is a single quantity that reflects changes in the degree of peripheral airway obstruction and closely correlates with R5 − R20. Normal ALX is generally <0.33 kPa/L (<3.37 cmH_2_0/L) [10]. A preliminary study with the MostGraph-01 showed the details of these parameters (Fig. 1a, b). The mean of each FOT parameter was calculated during approximately five respiratory cycles, including the inspiratory and expiratory phases. This technique may be more helpful than spirometry in evaluating the respiratory differences between preoperative and postoperative status, because respiratory impedance can be measured without any effort by the patient and airway narrowing can be detected. Furthermore, assessing the relationships between preoperative spirometry results and pre- and postoperative FOT measurements may help determine whether preoperative spirometry results can predict postoperative deterioration of respiratory impedance.

This study therefore examined the effects of tracheal intubation, inhalational general anesthesia and mechanical ventilation on perioperative respiratory impedance measured using FOT. In addition, the relationships between respiratory function measured by spirometry and respiratory impedance measured by FOT were evaluated.

## Methods

The study protocol was approved by the Ethics Committee of Osaka Medical College (reference number 1252), and all participants provided written informed consent. This prospective observational study assessed a cohort of 30 patients scheduled for transurethral resection of a bladder tumor under general anesthesia. Patients diagnosed with COPD, according to the Global Initiative for Chronic Obstructive Lung Disease (GOLD) guidelines [16], were excluded, as were patients with a history or symptoms of asthma, such as cough or wheeze at rest, and patients who had taken oral steroids or had a respiratory tract infection or exacerbation within the previous 3 months. Respiratory resistance (R5, R20 and R5 − R20) and reactance (X5, Fres and ALX) parameters were measured using the MostGraph-01 on the day before surgery. FOT measurements were made on patients sitting with their necks in a comfortable neutral posture; patients also wore a nose clip, and their cheeks were supported firmly. Participants were instructed to breath normally at functional residual capacity level (tidal breathing) for 30 s [9]. Spirometry was performed within 1 week before surgery using a spirometer (System 21 device, Minato Ikagaku, Osaka, Japan). Parameters measured included vital capacity (VC), forced vital capacity (FVC) and forced expiratory volume in the first second (FEV1.0).

Anesthesia was induced with intravenous propofol 1.5 mg/kg, rocuronium 1 mg/kg, a continuous infusion of remifentanil at 0.4 μg/kg/min and inhaled sevoflurane 3.0 %. The trachea was intubated with a tube of internal diameter 7.0 mm for women and 8.0 mm for men (Portex, Smiths Medical, Tokyo, Japan). Anesthesia was maintained with inhaled sevoflurane 1.2 %, intravenous remifentanil 0.25 μg/kg/min and intravenous rocuronium 0.4 μg/kg/min. The lungs were ventilated with a 0.4 fraction of inspired oxygen; a tidal volume of 7 ml/kg predicted ideal body weight with a positive end-expiratory pressure (PEEP) of 5 cmH_2_O. If oxygen saturation measured by pulse oximetry was <97 %, the tidal volume was increased to 8.5 ml/kg and more PEEP was added. At the end of surgery, patients were administered 1000 mg intravenous acetaminophen for pain relief, followed by 1.5 mg/kg intravenous sugammadex. Tracheal suctioning was performed once or twice before patients were extubated. The tracheal tube was removed when patients could breathe spontaneously with sufficient tidal volume and were able to communicate. Supplementary oxygen at 6 l/min was administered by a face-mask immediately after extubation. Respiratory impedance was measured within minutes after extubation using the MostGraph-01, with the patients in the Farrar position (sitting position with 45–50° head up-tilt).

Pre- and postoperative FOT measurements were compared, as were the relationships between those parameters and preoperative spirometry results. Postoperative FOT measurements were not performed in patients who became restless or nauseated.

This study was registered as a clinical study in a Japanese official clinical trial registry (JMACCT). The trial registration number was JMA-IIA00136.

### Statistical analysis

Patient characteristics were expressed as median with interquartile range (IQR). Pre- and postoperative respiratory impedance measurements were compared using Wilcoxon paired rank tests, with *p* < 0.01 considered statistically significant. The relationships between spirometry findings and respiratory impedance were assessed using Spearman’s rank correlation, with *p* < 0.01 considered statistically significant. All statistical analyses were performed using GraphPad Prism 6 software (GraphPad Software, La Jolla, CA).

## Results

Postoperative FOT measurements could not be made in four patients because of restlessness in three and nausea in one; therefore, 26 patients were included in the final analysis. None of these patients experienced any serious perioperative events and none required administration of atropine. Oxygen saturation remained >97 % in all patients. The characteristics of the 26 patients included in the analysis are shown in Table 1.

**Table 1:** Baseline demographic and clinical characteristics of the patient cohort

Preoperative parameter	*N* = 26
Male	23 (88.5 %)
Age (years)	68 (55 to 74)
Height (cm)	163.5 (160.6 to 168.9)
Weight (kg)	62.1 (56.8 to 69.1)
Body mass index (kg/m^2^)	23.7 (21.0 to 25.8)
Body surface area (m^2^)	1.7 (1.6 to 1.7)
Non-smoker	4 (15.4 %)
Smoker	5 (19.2 %)
Ex-smoker	17 (65.4 %)
Brinkman index	630 (300 to 1105)
VC (L)	3.1 (2.8 to 3.8)
%VC (% predicted)	101.4 (87.2 to 112.5)
FVC (L)	3.0 (2.7 to 3.8)
%FVC (% predicted)	98 (86.8 to 111.5)
FEV1.0 (L)	2.3 (1.7 to 2.7)
%FEV1.0 (% predicted)	74.3 (67.6 to 80.4)
FEV1.0/FVC ratio (%)	74.3 (67.6 to 80.4)
Anesthetic time (min)	89 (73 to 99)
Operating time (min)	47 (35 to 56)
Intravenous fluid volume (ml)	650 (588 to 700)

Data are expressed as median (interquartile range) or as number (percent). Brinkman index, defined as the number of cigarettes smoked per day multiplied by smoking years, was calculated in smokers and ex-smokers only. Preoperative spirometry data were measured within 1 week before surgery

Abbreviations: *VC* vital capacity, *FVC* forced vital capacity, *FEV1.0* forced expiratory volume in the first second

**Fig. 2:**
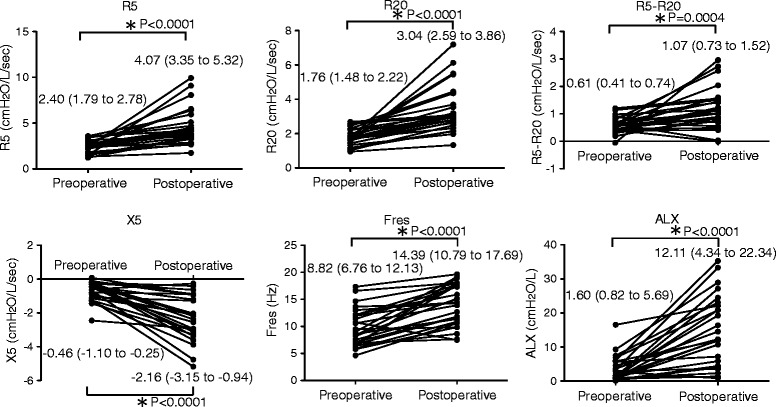
Comparison of pre- and postoperative respiratory impedance. All respiratory resistance components (R5, R20 and R5 − R20) increased significantly. Of the reactance components, X5 decreased and Fres and ALX increased significantly. All values are expressed as median with interquartile range

The median (IQR) of preoperative R5, R20, R5 − R20, X5, Fres and ALX were 2.40 (1.79 to 2.78) cmH_2_O/L/s, 1.76 (1.48 to 2.22) cmH_2_O/L/s, 0.61 (0.41 to 0.74) cmH_2_O/L/s, −0.46 (−1.10 to −0.25) cmH_2_O/L/s, 8.82 (6.76 to 12.13) Hz, and 1.60 (0.82 to 5.69) cmH_2_O/L, respectively. Postoperative R5, R20, R5 − R20, X5, Fres and ALX were 4.07 (3.35 to 5.32) cmH_2_O/L/s, 3.04 (2.59 to 3.86) cmH_2_O/L/s, 1.07 (0.73 to 1.52) cmH_2_O/L/s, −2.16 (−3.15 to −0.94) cmH_2_O/L/s, 14.39 (10.79 to 17.69) Hz and 12.11 (4.34 to 22.34) cmH_2_O/L, respectively. All components of resistance deteriorated significantly over the course of surgery, with median increases in R5, R20, and R5–R20 of 1.67 cmH_2_O/L/s (*p* < 0.0001), 1.28 cmH_2_O/L/s (*p* < 0.0001) and 0.46 cmH_2_O/L/s (*p* = 0.0004), respectively. The components of reactance also deteriorated significantly, with X5 decreasing 1.7 cmH_2_O/L/s (*p* < 0.0001), Fres increasing 5.57 Hz (*p* < 0.0001) and ALX increasing 10.51 cmH_2_O/L (*p* < 0.0001) (Fig. 2).

The relationships between preoperative spirometry and preoperative FOT findings are shown in Table 2. The preoperative predicted vital capacity (%VC) correlated significantly with X5 (*p* = 0.0013), Fres (*p* = 0.0001) and ALX (*p* = 0.0008). In addition, predicted forced expiratory volume in 1 s (%FEV1.0) correlated significantly with X5 (*p* = 0.0058), Fres (*p* = 0.0022) and ALX (*p* = 0.0030). Moreover, predicted forced vital capacity (%FVC) correlated significantly with X5 (*p* = 0.0033), Fres (*p* = 0.0012) and ALX (*p* = 0.0022).

**Table 2:** Relationships between spirometry and preoperative FOT findings

	%VC		%FEV1.0		%FVC		FEV1.0/FVC ratio	
	Coefficient	*p* value	Coefficient	*p* value	Coefficient	*p* value	Coefficient	*p* value
Preoperative R5 (cmH_2_O/L/s)	−0.371	0.0623	−0.381	0.0551	−0.319	0.1129	−0.222	0.2751
Preoperative R20 (cmH_2_O/L/s)	−0.326	0.1036	−0.338	0.0915	−0.294	0.1455	−0.167	0.4139
Preoperative R5-R20 (cmH_2_O/L/s)	−0.268	0.1855	−0.340	0.0897	−0.191	0.3505	−0.354	0.0758
Preoperative X5 (cmH_2_O/L/s)	0.595	0.0013*	0.526	0.0058*	0.555	0.0033*	0.176	0.3911
Preoperative Fres (Hz)	−0.642	0.0001*	−0.573	0.0022*	−0.599	0.0012*	−0.227	0.2640
Preoperative ALX (cmH_2_O/L)	−0.615	0.0008*	−0.558	0.0030*	−0.573	0.0022*	−0.221	0.2790

Abbreviations: *R5* respiratory resistance at 5 Hz, *R20* respiratory resistance at 20 Hz, *X5* respiratory reactance at 5 Hz, *Fres* resonance frequency, *ALX* area of low respiratory reactance, *VC* vital capacity, *FVC* forced vital capacity, *FEV1.0* forced expiratory volume in the first second. *P < 0.01

Statistically significant relationships were also observed between the same three preoperative spirometry parameters (%VC, %FEV1.0 and %FVC) and postoperative respiratory reactance (X5, Fres and ALX), as shown in Table 3. Preoperative predicted vital capacity (%VC) correlated significantly with X5 (*p* = 0.0002), Fres (*p* < 0.0001) and ALX (*p* < 0.0001). %FEV1.0 also correlated significantly with X5, Fres and ALX (*p* < 0.0001 each), and %FVC correlated significantly with X5 (*p* = 0.0001), Fres (*p* < 0.0001) and ALX (*p* < 0.0001).

**Table 3:** Relationships between spirometry and postoperative FOT findings

	%VC		%FEV1.0		%FVC		FEV1.0/FVC ratio	
	Coefficient	*p* value	Coefficient	*p* value	Coefficient	*p* value	Coefficient	*p* value
Postoperative R5 (cmH_2_O/L/s)	−0.285	0.1585	−0.290	0.1513	−0.262	0.1968	−0.097	0.6358
Postoperative R20 (cmH_2_O/L/s)	−0.180	0.3785	−0.283	0.1617	−0.171	0.4028	−0.197	0.3358
Postoperative R5-R20 (cmH_2_O/L/s)	−0.437	0.0256	−0.320	0.1113	−0.396	0.0450	0.029	0.8866
Postoperative X5 (cmH_2_O/L/s)	0.672	0.0002*	0.720	<0.0001*	0.684	0.0001*	0.336	0.0935
Postoperative Fres (Hz)	−0.742	<0.0001*	−0.708	<0.0001*	−0.741	<0.0001*	−0.229	0.2602
Postoperative ALX (cmH_2_O/L)	−0.699	<0.0001*	−0.734	<0.0001*	−0.706	<0.0001*	−0.335	0.0939

Abbreviations: *R5* respiratory resistance at 5 Hz, *R20* respiratory resistance at 20 Hz, *X5* respiratory reactance at 5 Hz, *Fres* resonance frequency, *ALX* area of low respiratory reactance, *VC* vital capacity, *FVC* forced vital capacity, *FEV1.0* forced expiratory volume in the first second. *P < 0.01

## Discussion

This study, which compared preoperative and postoperative measures by FOT, found that respiratory impedance deteriorated dramatically after anesthesia. To our knowledge, this is the first study to report postoperative FOT measurements immediately after anesthesia. All three components of Rrs (R5, R20 and R5-R20) increased. Assessments of Xrs showed that X5 decreased while Fres and ALX increased. The deterioration in respiratory impedance increased with a longer period of anesthesia [11]. Anesthesia and mechanical ventilation reduce respiratory compliance, increase Rrs, and cause atelectasis and airway closure, resulting in shunt [17]. This study, however, focused on perioperative changes in respiratory impedance, thought to be caused by: 1) edema of the vocal folds, resulting from endotracheal intubation; 2) proximal and peripheral airway edema, resulting from administration of excess intravenous fluid and/or the Trendelenburg position; 3) sputum (i.e. mucous secreted by the lungs, bronchi and trachea that can include oral discharge); 4) asthma; and/or 5) ventilator-induced lung injury (VILI) [18]. In this study, patients were in the lithotomy position without head-down tilt during surgery. Moreover, the volume of intraoperative intravenous fluid administered was not regarded as excessive because the operation time was relatively short. We also adopted a lung protection strategy for mechanical ventilation by selecting a tidal volume of 7 ml/kg and a PEEP of 5 cmH_2_O to minimize lung injury [18, 19], although the I:E ratio in all patients was 1:2 [20]. Nonetheless, in our cohort, deterioration in respiratory function was likely caused by a combination of these factors, including very mild VILI during surgery in the Trendelenburg position and stimulation by intubation, extubation, and tracheal suctioning. The factor having the greatest influence was unclear. A study using endotracheal intubation and a subglottic device for airway management may determine the influence of tracheal intubation and extubation, whereas a study with or without endotracheal suctioning may determine the influence of stimulation by suctioning. Use of a tracheal tube and endotracheal suctioning can cause coughing, although all patients probably experienced very mild VILI caused by mechanical ventilation with high and/or low lung volume. Our findings suggest that measuring Rrs can help to understand respiratory compliance during mechanical ventilation and to detect VILI caused by overdistension of the lungs and/or atelectrauma. A comparison of Rrs before and after alveolar recruitment maneuvers (ARMs) can determine the effect of ARM on VILI. Recommended ARM generally consists of holding the lungs at 40 cmH_2_O for more than 7–8 s during mechanical ventilation [21]. Rrs can be measured only during the expiratory phase of volume-controlled ventilation under anesthesia, with a simple modification of the FOT speaker of MostGraph-01. It may not be possible, however, to correctly measure Xrs during mechanical ventilation.

We observed that the components of Xrs correlated with %VC and %FEV1.0. Low %VC indicates a restrictive lung disorder, whereas low %FEV1.0 indicates an obstructive disorder. Reductions in X5 and increases in Fres and ALX are expected when respiratory compliance decreases. FOT has been reported to be more sensitive than spirometry in detecting obstructive pulmonary disease and in assessing the effects of bronchodilators [22–25]. However, because of difficulties interpreting Xrs, we could not clearly determine the relationships between spirometry and FOT measurements.

Changes in respiratory function were measured after transurethral bladder tumor resection under general anesthesia. This operation was chosen because operating time is relatively brief and predictable, there is little postoperative pain, patients are able to sit up relatively quickly afterwards, there is little blood loss or fluid shift, and surgery does not affect ventilatory strategy. The main advantages of FOT are that minimal cooperation is required from the patients and no respiratory maneuvers are needed. Therefore, respiratory impedance should be measured whenever spirometry cannot be performed or would likely be unreliable. In addition, although the MostGraph-01 recording technique is well established, the extent of cheek support may influence respiratory impedance, as it can affect upper airway artifacts [26].

Future studies should focus on establishing the time course of postoperative recovery of respiratory impedance and the effects of treatments. FOT can also be used to determine the need for postoperative interventions and physical therapy in severe cases. Furthermore, differences in respiratory impedance measured during the inspiratory and expiratory phases may help illuminate the underlying mechanisms [23–27]. The influence of anesthesia on respiratory impedance in patients with peripheral airway inflammation should also be assessed. Measurement of fractional exhaled nitric oxide, a surrogate marker of eosinophilic airway inflammation [24, 25, 28, 29], may also enhance understanding of the relationships between biochemical and physiologic influences on respiratory function.

This study had several limitations. First, it did not compare postoperative FOT measurements with postoperative spirometry measurements, because performing postoperative spirometry on the day of surgery carries a risk of bleeding from the bladder. Furthermore, the study design did not include standardization of the administration of fluids and use of bladder irrigation. Limiting fluid volume likely would not have affected our results, because transurethral resection of bladder tumors is of short duration and minimally invasive. Bladder irrigation was not determined. None of these patients experienced transurethral resection (TUR) syndrome or intra- or postoperative hypoxia. However, a surgical procedure that does not have to assess the influence of fluid shifts is ideal for investigating respiratory mechanisms. Another limitation of our study was the relatively small sample size. Further studies are needed to better evaluate changes in perioperative respiratory mechanics.

## Conclusions

All parameters of respiratory resistance and reactance deteriorated significantly after general anesthesia and mechanical ventilation. Components of resistance (R5, R20 and R5-R20) increased, while, of the reactance components, X5 decreased and Fres and ALX increased. Pre- and postoperative respiratory reactance correlated with preoperative pulmonary function measured by spirometry. Measuring FOT with MostGraph-01 is a straightforward means of measuring perioperative changes in respiratory impedance and has the potential to determine respiratory compliance effected by general anesthesia.

## Abbreviations

%FEV1.0, predicted percent forced expiratory volume in the first second; %FVC, percent forced vital capacity; %VC, predicted percent vital capacity; ALX, area of low reactance; COPD, chronic obstructive pulmonary disease; FEV1.0, forced expiratory volume in one second; FOT, forced oscillation technique; Fres, resonant frequency; FVC, forced vital capacity; IQR, interquartile range; PEEP, positive end-expiratory pressure; R20, respiratory resistance at 20Hz; R5 − R20, difference between R5 and R20; R5, respiratory resistance at 5Hz; Rrs, respiratory resistance; VC, vital capacity; VILI, ventilator induced lung injury; X5, reactance at 5Hz.; Xrs, respiratory reactance
